# Pilot‐Scale Testing of Blessing/Biofield Energy Treatment (BET) for Improving Eggs and Meat Quality of Rhode Island Red Laying Hens

**DOI:** 10.1002/vms3.70798

**Published:** 2026-01-19

**Authors:** Mahendra Kumar Trivedi, Vaibhav Rajan Parulkar, Dahryn Trivedi, Alice Branton, Sambhu Mondal, Snehasis Jana

**Affiliations:** ^1^ Trivedi Global, Inc. Research and Development Henderson Nevada USA; ^2^ Divine Connection International Southlake Texas USA; ^3^ Trivedi Science Research Laboratory Pvt. Ltd. Research and Development Thane Maharashtra India

**Keywords:** blessing energy treatment, egg quality, meat quality, morphological parameter, Rhode Island Red hens, sensory

## Abstract

**Background:**

Rising feed costs and concerns about poultry supplements have sparked interest in natural, complementary and alternative methods to enhance poultry productivity.

**Objective:**

This pilot study aimed to compare the growth and quality of meat and eggs from blessing/biofield energy‐treated Rhode Island Red (RIR) hens with those from untreated control hens.

**Methods:**

Fifty 18‐week‐old RIR laying hens were divided into two groups: control (CONHG; *n* = 25) and treated (BETHG; *n* = 25). We assessed egg‐laying performance, quality, nutritional content, carcass characteristics, sensory attributes and microbial analysis.

**Results:**

The BETHG group showed significant improvements in both external and internal egg quality parameters (specifically, egg weight, height, diameter, albumen weight, albumen height, yolk weight, yolk height and yolk index) compared to the CONHG group. The egg‐laying rate and edible meat weight increased significantly (*p* ≤ 0.001) by 28.38% and 40.95%, respectively, in BETHG. Cholecalciferol (D_3_) levels in BETHG increased by 140.36% (meat) and 160.27% (eggs), while iron levels rose by 73.92% (meat) and 95.17% (eggs). Zinc levels were significantly higher in BETHG by 122.39% (meat; *p* = 0.002) and 70.81% (eggs; *p* ≤ 0.001). Additionally, linoleic acid (C18:2) increased by 383.33% (meat; *p* ≤ 0.001) and alpha‐linolenic acid (C18:3) by 166.67% (eggs; *p* = 0.024). In the BETHG eggs, lutein improved by 100% (*p* = 0.002) and cis‐zeaxanthin enhanced by 87.5% (*p* = 0.035) relative to the CONHG group. Sensory characteristics in both meat and eggs improved significantly (*p* ≤ 0.001) in the BETHG group compared to the CONHG group.

**Conclusion:**

Blessing/biofield energy treatment significantly enhanced the quality and nutritional value of both meat and eggs compared to control group.

## Graphical abstract

This pilot study established that Blessing (biofield) Energy Treatment significantly enhanced the quality and nutritional value of both meat and eggs of 28‐week‐old RIR laying hens compared to the control. A graphical abstract summarising the study design and outcomes of this study is provided as a separate file (Graphical_Abstract.jpeg).

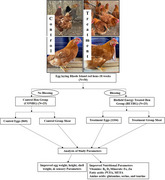

**Abbreviations**
BETHG
: biofield energy‐treated hen group
CONHG
: control hen group
FCR: feed conversion ratio
RIR: Rhode Island Red

## 1 Introduction

Rhode Island Red (RIR) is a brown‐egg‐laying, dual‐purpose chicken breed. RIR is not a hybrid nor an indigenous bird, rather than a crossbreed between Asian birds and brown leghorn (Isa et al. 2020). Chicken meat and eggs are rich sources of proteins necessary for human health (Beski et al. 2015). Beyond its proteins, chicken has relatively low fat (without skin), high levels of unsaturated fats, is lower in cholesterol and is packed with essential nutrients (Geiker et al. 2021; Marangoni et al. 2015). The chicken meat is very popular, including its ease of preparation, consistency in quality and availability in prepackaged, branded, raw and ready‐to‐eat preparations (Geiker et al. 2021). Although the ratio of fat to muscle in the edible parts can vary depending on the species and the nutrient content of muscle. The quality of animal fat and the amount of nutrients are primarily determined by the animal's diet and genetics (National Research Council (US) Board on Agriculture and Renewable Resources 1976). The hen egg stands out as an excellent and naturally abundant food source, making a significant contribution to global human diets. The eggs consisted of around 60% white, 30% yolk and 10% shell, which translates chemically into 75% water, 12% protein and 12% lipid (Wasielewska 2021). The product is nutrient‐dense and economical, making it a key ingredient in a wide range of foods. There are many external factors that influence egg quality, including feed quality, hen breed and the type of feeding system used (Gao et al. 2021). Consequently, a significant body of current research is dedicated to modifying egg composition through strategic alterations in feed components, feeding strategies and even breed selection. While the influence of feed on fatty acid alteration in eggs has been a primary area of study (Grobas et al. 2001), increasing attention is now being paid to enriching eggs with essential vitamins, minerals, amino acids and beneficial carotenoids (Leeson and Caston 2003; Sari et al. 2002).

In this context, it is assumed that complementary and alternative medicine therapy like blessing (biofield) energy treatment would improve the nutritional quality of eggs and meats. The Trivedi Effect is a unique and inherent biofield energy therapy approach. Globally renowned religious and spiritual leader who can harness Divine Energy/the Grace of God from the environment or universe and transmit it to any living organism or non‐living material(s) around the globe. The efficacy of Blessing/Biofield Energy Treatment (Trivedi Effect) has been published in numerous peer‐reviewed science journals with significant outcomes in both preclinical (Trivedi and Tallapragada 2009; M. K. Trivedi et al. 2015; D. Trivedi et al. 2025a; M. K. Trivedi et al. 2025b) and clinical studies (Branton et al. 2025; M. K. Trivedi et al. 2023; M. K. Trivedi et al. 2024b; D. Trivedi, Trivedi, et al. 2025).

The idea of this study is based on the potential impact of Trivedi Effect on livestock, specifically on poultry (M. K. Trivedi et al. 2015; M. K. Trivedi et al. 2025b; D. Trivedi et al. 2025a), and the lack of complementary and alternative treatment approaches like biofield energy treatment that affect laying hen performance remains underexplored. Therefore, this study was planned to evaluate the impact of the Trivedi Effect (blessing energy treatment) on the quality analysis of eggs and meat of RIR laying hens with respect to the control hens. The various eggs and meat‐quality‐related parameters, like albumen height, albumen weight, albumen diameter, albumen index, yolk height, yolk diameter, yolk weight, yolk index and Haugh unit, egg weight, egg height, egg diameter, egg shape index, eggshell weight and eggshell thickness, etc., and nutritional parameters, namely, vitamins, minerals, free amino acids, free fatty acid profiles, carotenoids, protein content in egg yolk and egg shells, sensory parameters and microbial analysis, etc., were measured.

## 2 Materials and Methods

### 2.1 Selection of Breeds and Study Design

**FIGURE 1:**
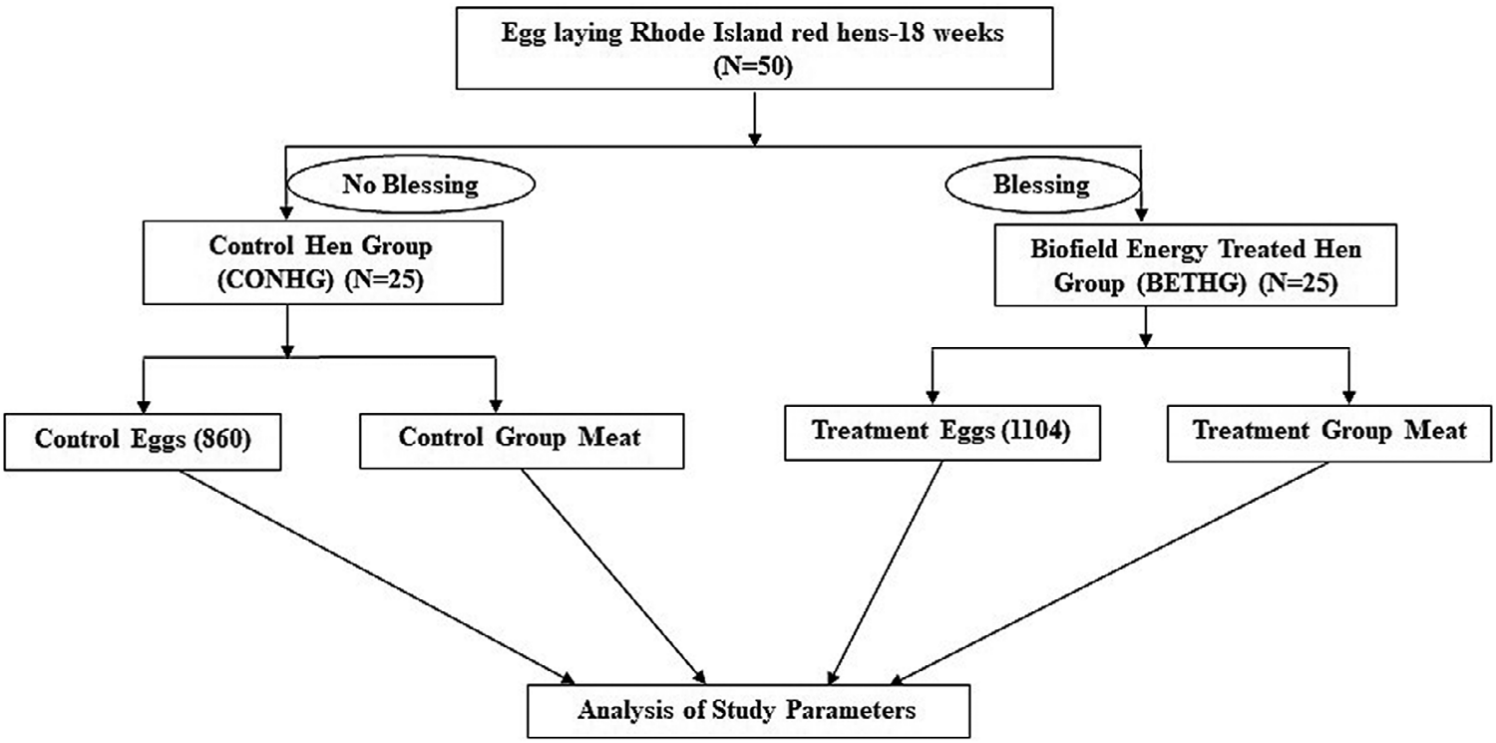
The schematic details of the test system allocation and study design.

Commercially available 18‐week‐old RIR laying hens (*n* = 50) were used in this study as test system. Fifty hens were equally divided into two groups; one group was defined as the unblessed/control RIR hen group (CONHG) (*n* = 25), where no treatment was given, and another one referred to as the biofield (blessing) energy‐treated RIR hen group (BETHG) (*n* = 25), where blessing therapy was given by renowned blessing practitioner. Detailed design is shown in Figure 1.

### 2.2 Farm Arrangement

Two different sheds or areas (spaces) in a shed were designated for the control and biofield energy treatment groups. The space allocated was approximately 2 square feet per bird. An adequate feeder and water drinker were placed to provide sufficient water and feed to the hens. All the birds were provided with free access to clean drinking water, which was manually replenished around the clock. All the hens were reared naturally in a deep litter system for the entire study period up to the appropriate age of slaughter.

### 2.3 Blessing (Biofield) Energy Treatment Strategy

The RIR laying hens (18‐week‐old) were divided into two groups, that is, control and blessing (biofield) energy treatment groups. The control group did not receive any treatment, and the blessing energy treatment group received *in‐person* (physical presence) blessing/prayer energy treatment for approximately 4 min by an experienced (> 15 years) renowned spiritual energy healing practitioner at the farm.

### 2.4 Feed Supply

All the RIR hens (control and Blessing energy‐treated) were fed with standard commercial layer mash feed obtained from Leland Milling Company, Utah (UT), USA, throughout the laying period. The feed ingredients and analysis of essential components, as shown in Table 1.

**TABLE 1:** Essential components of 18%‐layer mash.

Feed analysis	
Component	Quantity
Crude protein (min)	18%
Lysine (min)	1.3%
Methionine (min)	0.37%
Crude fat (min)	4%
Crude fiber (max)	6%
Calcium (min/max)	0.35%/0.40%
Phosphorus (min)	0.42%
Salt (min/max)	0.54/0.55%
Vitamin A (min)	2550 IU/LB
Selenium (min)	0.17 PPM
Ingredient	

*Note*: Chopped yellow corn, extruded soybean meal, processed grain by‐products, monocalcium phosphate, dicalcium phosphate, choline chloride, ground limestone, sodium bentonite, zinc sulphate, manganese sulphate, mineral oil, ferrous sulphate, niacin supplement, selenium yeast, ethoxyquin (a preservative), vitamin E supplement, copper sulphate, D‐calcium pantothenate, vitamin A acetate, vitamin D supplement, ethylenediamine dihydroiodide, riboflavin supplement, biotin, pyridoxine hydrochloride, menadione nicotinamide bisulfite, thiamine mononitrate, vitamin B_12_ supplement, folic acid, sodium selenite.

Abbreviations: IU, International units; LB, pound; Max, maximum; Min, minimum; PPM, parts per million.

### 2.5 Detailed Procedure

#### 2.5.1 Monitoring and Measurements

The hens were monitored daily by the supervisor for signs and symptoms, health status and mortality. Six hens, four dozen of eggs (for nutritional parameters), and two dozen of eggs (physical egg quality parameters) were used from each group to monitor the defined parameters in this study. The feed was withdrawn for 12 h before the day of slaughtering.

### 2.6 Effects of Blessing Treatment on Egg Quality Characteristics

Hens were weighed at the end of the experiment. During the experiment, feed intake was recorded, while all eggs, their individual weights and the number of eggs were recorded daily. Egg‐laying rate (%) and feed conversion ratio (FCR) were calculated as feed consumed divided by egg mass. Of the eggs produced during the last 6 week randomly selected eggs were used to determine certain external (egg weight, egg height, egg diameter, egg shape index, eggshell weight and eggshell thickness), internal (albumen height, albumen weight, albumen diameter, albumen index, yolk height, yolk diameter, yolk weight, yolk index and Haugh unit), nutritional and sensory parameters. The weights of egg, egg yolk, albumen and eggshell were measured with the help of a digital scale. The egg height, egg diameter, albumen height, albumen diameter, yolk height and yolk diameter were measured using the Vernier calliper scale. Egg shell thickness was measured using micrometer screw gauge. Egg shape index was calculated as egg diameter divided by egg length (Onimisi et al. 2012). The albumen index was calculated as albumen height divided by albumen diameter. The yolk index was calculated as yolk height divided by yolk diameter. Haugh unit was determined using the USDA interior egg quality measure.

$$\mathrm{Hu}=100\;\log\;\left(h-1.7w^{0.37}+7.6\right)$$

where Hu is the Haugh unit, h is the observed height of the albumen in mm and w is the weight of egg in g.

### 2.7 Slaughtering and Dressing

After taking the live weight of each bird, operations of hygienic slaughtering and poultry dressing were initiated. Exsanguinations were done by manually cutting the carotid arteries. After scalding (52°C–54°C, 4 min), the carcasses were defeathered and eviscerated, and the meat inspection was conducted. The weight of each dressed carcass was recorded.

### 2.8 Sampling for Meat and Egg Quality Evaluation

The different morphological and nutritional content was analysed in the meat and egg samples, such as vitamins, minerals, free amino acids, fats, free fatty acid profiles, sensory parameters and carotenoids. Additionally, protein and microbial analyses on meat, eggs, egg yolk and egg shells were also analysed. All the parameters except sensory were analysed at the end of the study as per standard procedure by Eurofins Microbiology Laboratories, Texas, USA.

### 2.9 Sensory/Organoleptic Evaluation of Meat and Eggs

Sensory assessment was performed according to Parpinello et al. (2006), with few modifications.

#### 2.9.1 Meat Preparation

Pieces of RIR meat were selected randomly, ensuring uniformity in size and weight (approximately 1250 g per sample). The meat was cut into standardised pieces (e.g., 2–3 cm thick slices or cubes) to ensure consistent cooking. A pot containing approximately 3000 mL of water was brought to a boil for approximately 30 min, and salt was added for seasoning. Cooking continued for another 5 min or until the meat was tender and could be easily separated with a fork. Cooked meat samples were allowed to rest for 2–3 min before evaluation.

#### 2.9.2 Egg Preparation

Twelve eggs from each group were boiled at same condition in a kettle for 10 min. Then, they were cooled, peeled, had the yolks separated and were presented to the consumers for evaluation of various parameters.

## 3 Assessment

A consumer preference test was conducted with 20 consumers who were asked to express their impressions of the coded groups of meat samples. Consumers represented diverse age groups, socioeconomic status and educational qualifications. Coded samples of cooked breast and thigh meat were provided to consumers for tasting, and their scores were noted. Based on six vital parameters, namely, colour, flavour, odour, juiciness, tenderness and quality/acceptability, a sensory evaluation was conducted to assess the quality of cooked chicken samples using 9‐point hedonic scale (9: extremely, 8: very much, 7: moderately, 6: slightly, 5: neither like nor dislike, 4: dislike slightly, 3: dislike moderately, 2: dislike very much, 1: dislike extremely) to assess the scores (Wichchukit and O'Mahony 2015). Similarly, the consumers were asked to provide scores of colour, flavour, taste, mouthfeel, aroma and overall acceptance of each egg sample on 9‐point hedonic scale (Wichchukit and O'Mahony 2015).

### 3.1 Statistics

Data were expressed as mean ± SEM. The study data were analysed using Student's *t*‐ test. *p* < 0.05 was considered the level of statistical significance.

## 4 RESULTS

### 4.1 Evaluation of Signs, Symptoms and Mortality

**FIGURE 2:**
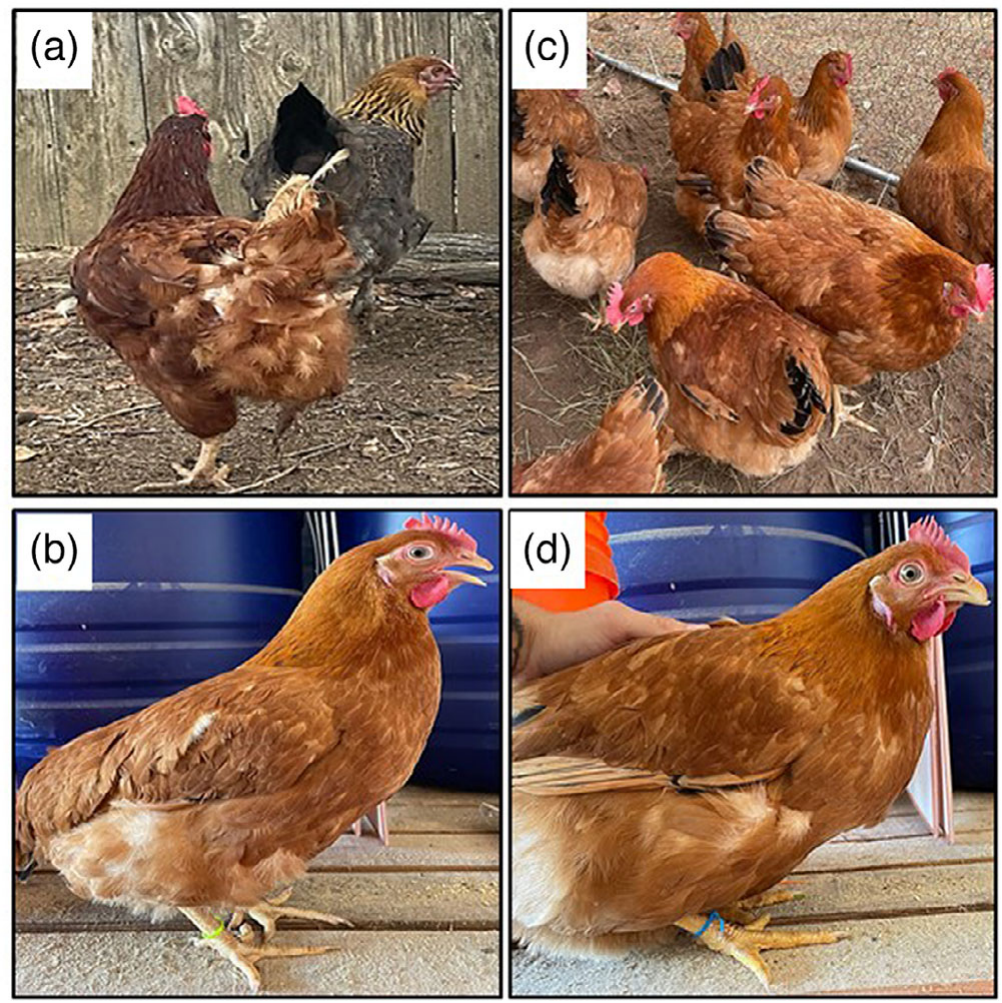
Representative photographs of untreated/control Rhode Island Red (RIR) laying hens (A and B) and blessing/biofield energy‐treated RIR laying hens (C and D).

No study‐related signs, symptoms, or mortality were observed in either the control (CONHG) or blessing (biofield) energy treatment group (BETHG). Some representative photographs, as shown in Figure 2, indicated the visual differences between the CONHG and BETHG. The BETHG looked bigger in size and about 34% heavier, with very vibrant, shiny and smooth feathers, very strong muscles and very healthy compared to the CONHG. Moreover, the BETHG showed more calmness, no anxiety, no fighting with each other or picking at each other's feathers, and also less smell with respect to the CONHG.

### 4.2 Effects of Blessing (Biofield) Treatment on Egg Quality Characteristics

Both external and internal parameters of blessing energy‐treated eggs were improved, as shown in Table 2. External parameters such as egg weight, height and diameter were significantly increased by 17.33% (*p* ≤ 0.001), 6.10% (*p* = 0.024) and 8.2%, respectively, in the blessing energy treated hens’ group (BETHG) compared to the CONHG. Egg shell weight was significantly (*p* = 0.019) increased by 5.28% in the BETHG compared to the CONHG. Similarly, internal parameters of eggs such as albumen weight, height, and diameter were significantly increased by 11.10% (*p* ≤ 0.001), 7.35% (*p* = 0.030) and 12.03% (*p* ≤ 0.001), respectively, in the BETHG compared to the CONHG. The yolk weight, height and diameter were significantly increased by 25.53% (*p* ≤ 0.001), 40% (*p* ≤ 0.001) and 6.31% (*p* = 0.018), respectively, in the BETHG compared to the CONHG. Yolk index was increased by 31.67% in the BETHG compared to CONHG (Table 2).

**TABLE 2:** Effects of Blessing (Biofield) energy treatment on egg quality of Rhode Island Red (RIR) hens.

Parameter	CONHG	BETHG
External		
Egg weight (g)	53.16 ± 1.15	62.37 ± 1.13***
Egg height (cm)	5.41 ± 0.10	5.74 ± 0.10*
Egg diameter (cm)	4.27 ± 0.04	4.62 ± 0.17
Egg shape index (%)	78.93	80.49
Egg shell weight (g)	5.87 ± 0.09	6.18 ± 0.09*
Egg shell thickness (mm)	0.48 ± 0.02	0.50 ± 0.01
Internal		
Albumen weight (g)	30.26 ± 0.25	33.62 ± 0.25***
Albumen height (cm)	0.68 ± 0.02	0.73 ± 0.01*
Albumen diameter (cm)	6.98 ± 0.17	7.82 ± 0.10***
Albumen index (%)	9.74	9.34
Yolk weight (g)	14.22 ± 0.21	17.85 ± 0.17***
Yolk height (cm)	1.25 ± 0.07	1.75 ± 0.02***
Yolk diameter (cm)	4.12 ± 0.07	4.38 ± 0.08*
Yolk index (%)	30.34	39.95
Haugh unit (HU)	84.39	84.70

*Note*: CONHG: Control RIR hens; BETHG: Blessing/Biofield treated RIR hens; *p ≤ 0.05 and ***p ≤ 0.001 with respect to CONHG. Data were represented as mean ± SEM (*n* = 24). Statistical analysis was performed using Student *t*‐test. Egg shape index = (Egg diameter/Egg height) x 100; albumen index = (Albumen height/albumen diameter) x 100; yolk index = (Yolk height/yolk diameter) x 100.

Feed intake and FCR were significantly reduced by 5.78% (*p* = 0.012) and 22.38% (*p* ≤ 0.001), respectively, in the BETHG, compared to the CONHG. Egg laying rate was significantly (*p* ≤ 0.001) increased by 28.38% in the BETHG, compared to the CONHG. The slaughter weight was significantly (*p* ≤ 0.001) increased by 34.66% in the BETHG compared to the CONHG. Skin, feather and internal organs’ weight were improved in the BETHG, compared to CONHG. Edible meat weight was significantly (*p* ≤ 0.001) increased by 40.95% in the BETHG compared to the CONHG (Table 3).

**TABLE 3:** Effects of blessing/biofield treatment on the egg's performance and carcass parameters.

Parameter	CONHG	BETHG
Egg's performance		
Feed intake (g/day/hen)	112.31 ± 1.35	105.82 ± 1.65*
Egg laying rate (%)	76.44 ± 0.27	98.13 ± 0.39***
FCR (feed intake: egg weight)	2.10 ± 0.03	1.63 ± 0.05***
Carcass parameter		
Farm/slaughter weight (kg) at 28^th^ week	1.76 ± 0.04	2.37 ± 0.05***
Feather + skin weight (kg)	0.46 ± 0.02	0.49 ± 0.02
Internal all organs weight (kg)	0.29 ± 0.01	0.32 ± 0.01
Edible meat weight (kg)	1.05 ± 0.03	1.48 ± 0.08***

*Note*: CONHG: Control Rhode Island Red (RIR) hens; BETHG: Blessing/Biofield treated RIR hens; *p ≤ 0.05 and ***p ≤ 0.001 with respect to CONHG. Data were represented as mean ± SEM (*n* = 6). Statistical analysis was performed using Student *t*‐test.

Abbreviation: FCR, feed conversion ratio.

### 4.3 Evaluation of Vital Nutrients in RIR Meat

Vitamins such as B_9_ and D_3_ were significantly (*p* ≤ 0.001) increased by 100% and 140.36%, respectively, in the BETHG compared to the CONHG. Other vitamins such as B_5_, B_12_ and choline were increased non‐significantly in the BETHG compared to the CONHG. Minerals such as iron (Fe), zinc (Zn) and sodium (Na) were significantly increased by 73.92% (*p* ≤ 0.001), 122.39% (*p* = 0.002) and 50.46% (*p* ≤ 0.001), respectively, in the BETHG compared to the CONHG. Copper was increased by 48.72% non‐significantly in the BETHG compared to the CONHG. Saturated fatty acids such as palmitic acid (C16:0) and stearic acid (C18:0) were significantly elevated in the BETHG by 150% (*p* ≤ 0.001) and 120% (*p* = 0.002), respectively, compared to the CONHG. Unsaturated fatty acids such as oleic acid (C18:1) and linoleic acid (C18:2) were significantly increased by 255.56% (*p* = 0.029) and 383.33% (*p* ≤ 0.001), respectively, in the BETHG compared to the CONHG. The pull of total omega‐6 and nine isomers was significantly increased by 216.67% (*p* ≤ 0.001) and 220% (*p* = 0.010), respectively, in the BETHG compared to the CONHG. Total fatty acids, MUFA and PUFA were significantly (*p* ≤ 0.001) increased by 195.12%, 225% and 223.08%, respectively, in the BETHG compared to the CONHG. Amino acids such as glutamine, serine and taurine were significantly increased in the BETHG by 95% (*p* = 0.040), 76.92% (*p* = 0.005) and 146.67% (*p* ≤ 0.001), respectively, compared to the CONHG (Table 4).

**TABLE 4:** Evaluation of nutritional contents such as vitamins, minerals, fatty acids, and amino acids in Rhode Island Red (RIR) meat.

Parameter	CONHG	BETHG
Vitamin		
Vitamin B_9_/total folate (mg/100 g)	0.01 ± 0.00	0.02 ± 0.00***
Vitamin B_5_/pantothenic acid (mg/100 g)	1.03 ± 0.01	1.14 ± 0.12
Vitamin D_3_/cholecalciferol (IU/100 g)	13.90 ± 1.35	33.41 ± 1.92***
Vitamin B_12_/cobalamin (µg/100 g)	0.79 ± 0.06	1.19 ± 0.63
Total choline (mg/100 g)	52.10 ± 4.18	53.50 ± 2.81
Mineral		
Copper (ppm)	0.39 ± 0.05	0.58 ± 0.07
Iron (ppm)	4.87 ± 0.37	8.47 ± 0.14***
Zinc (ppm)	6.61 ± 0.23	14.70 ± 1.99**
Sodium (ppm)	436 ± 9.24	656 ± 6.93***
Fatty acid profiles		
C16:0 (Palmitic acid, %)	0.10 ± 0.00	0.25 ± 0.02***
C18:0 (Stearic acid, %)	0.05 ± 0.01	0.11 ± 0.01**
C18:1 Omega 9 (oleic acid, %)	0.09 ± 0.01	0.32 ± 0.09*
C18:2 Omega 6 (linoleic acid, %)	0.06 ± 0.01	0.29 ± 0.03***
C20:4 Omega 6 (arachidonic acid, %)	0.05 ± 0.01	0.06 ± 0.01
C20:4, Total (eicosatetraenoic acid, %)	0.05 ± 0.01	0.06 ± 0.01
Total omega 6 isomers (%)	0.12 ± 0.02	0.38 ± 0.05***
Total omega 9 isomers (%)	0.10 ± 0.00	0.32 ± 0.07**
Total monounsaturated fatty acids (MUFA, %)	0.12 ± 0.01	0.39 ± 0.02***
Total polyunsaturated fatty acids (PUFA, %)	0.13 ± 0.01	0.42 ± 0.02***
Total fatty acids (%)	0.41 ± 0.06	1.21 ± 0.08***
Cholesterol (mg/100 g)	57.30 ± 4.81	53.50 ± 6.24
Amino acids profile		
Glutamic acid (mg/g)	0.24 ± 0.02	0.25 ± 0.03
Glutamine (mg/g)	0.20 ± 0.01	0.39 ± 0.08*
Serine (mg/g)	0.13 ± 0.02	0.23 ± 0.02**
Hydroxyproline (mg/g)	1.02 ± 0.12	1.04 ± 0.10
Taurine (mg/g)	0.60 ± 0.06	1.48 ± 0.06***

*Note*: CONHG: Control Rhode Island Red hens; BETHG: Blessing/Biofield treated Rhode Island Red hens; *p ≤ 0.05, **p ≤ 0.01, and ***p ≤ 0.001 with respect to CONHG. Data were represented as Mean ± SEM (*n* = 3). Statistical analysis was performed using Student *t*‐test.

### 4.4 Evaluation of Essential Nutrients in Rhode Island Eggs

Vitamins such as E, D_3_ and A were significantly increased by 200% (*p* = 0.005), 160.27% (*p* ≤ 0.001) and 62.08% (*p* ≤ 0.001), respectively, in the BETHG compared to the CONHG. Other vitamin levels were increased in the BETHG compared to CONHG, although the data were not statistically significant. Minerals such as Ca, Fe, P, Zn and Se were significantly increased in the BETHG by 49.36% (*p* ≤ 0.001), 95.17% (*p* ≤ 0.001), 53.70% (*p* ≤ 0.001), 70.81% (*p* ≤ 0.001) and 20.05% (*p* = 0.009), respectively, compared to the CONHG. Other minerals were non‐significantly improved in the BETHG compared to CONHG. Essential fatty acids such as linoleic acid (C18:2/omega‐6), alpha‐linolenic acid (C18:3/omega‐3) and docosahexaenoic acid (C22:6/DHA) were significantly increased by 40.91% (*p* = 0.026), 166.67% (*p* = 0.024) and 150% (*p* = 0.013), respectively, in the BETHG compared to the CONHG. Total omega‐3 isomers were also significantly (*p* ≤ 0.001) improved by 137.5% in the BETHG compared to CONHG. Protein content in egg shell was significantly (*p* = 0.037) increased by 40.84% in the BETHG compared to the CONHG. Carotenoids such as lutein and cis‐zeaxanthin were significantly improved by 100% (*p* = 0.002) and 87.5% (*p* = 0.035), respectively, in BETHG compared to the CONHG (Table 5).

**TABLE 5:** Evaluation of nutritional content like vitamins, minerals, fatty acids, amino acids and carotenoids in Rhode Island Red (RIR) eggs.

Parameter	CONHG	BETHG
Vitamin		
Vitamin E‐gamma‐tocopherol (mg/100 g)	0.46 ± 0.12	1.38 ± 0.11**
Vitamin B_2_/riboflavin (mg/100 g)	0.44 ± 0.11	0.45 ± 0.14
Vitamin B_7_/biotin (mg/100 g)	0.03 ± 0.01	0.05 ± 0.01
Vitamin B_9_/total folate (mg/100 g)	0.10 ± 0.05	0.14 ± 0.04
Vitamin K_2_ (as MK_4_) (µg/g)	0.06 ± 0.01	0.09 ± 0.01
Vitamin D_3_ (IU/100 g)	58.40 ± 3.90	152 ± 3.66***
Vitamin A/retinol (IU/100 g)	356 ± 10.85	577 ± 10.18***
Vitamin B_12_/cobalamin (µg/100 g)	2.84 ± 0.35	3.05 ± 0.24
Total choline (mg/100 g)	294 ± 9.68	309 ± 9.29
Mineral		
Calcium (ppm)	314 ± 2.31	469 ± 5.77***
Copper (ppm)	0.39 ± 0.10	0.52 ± 0.03
Iron (ppm)	9.94 ± 0.18	19.40 ± 1.00***
Magnesium (ppm)	110 ± 3.46	116 ± 2.31
Manganese (ppm)	0.18 ± 0.02	0.27 ± 0.06
Phosphorus (ppm)	1080 ± 37.53	1660 ± 45.03***
Zinc (ppm)	6.44 ± 0.39	11.00 ± 0.27***
Selenium (ppb)	364 ± 11.55	437 ± 10.39**
Fatty acid profile		
C16:0 (Palmitic acid)	1.80 ± 0.2	1.97 ± 0.21
C16:1 Omega 7‐palmitoleic acid	0.14 ± 0.02	0.15 ± 0.02
C17:0 (Margaric acid)	0.03 ± 0.01	0.02 ± 0.01
C18:0 (Stearic acid)	0.63 ± 0.14	0.69 ± 0.05
C18:2 Omega 6 (linoleic acid)	1.10 ± 0.07	1.55 ± 0.11*
C18:3 Omega 3 (alpha‐linolenic acid)	0.03 ± 0.01	0.08 ± 0.01*
C18:3 Total (linolenic acid + isomers)	0.03 ± 0.01	0.09 ± 0.03
C20:4 Omega 6 (arachidonic acid)	0.14 ± 0.01	0.16 ± 0.01
C20:4 Total (eicosatetraenoic acid)	0.14 ± 0.01	0.16 ± 0.01
C22:6 Docosahexaenoic omega 3‐DHA	0.04 ± 0.01	0.10 ± 0.01*
Total omega 3 isomers	0.08 ± 0.01	0.19 ± 0.01***
Total omega 6 isomers	1.35 ± 0.07	1.81 ± 0.20
Total polyunsaturated fatty acids (PUFA)	1.44 ± 0.17	2.02 ± 0.14
Total trans fatty acids	0.06 ± 0.01	0.03 ± 0.01
Total fats (as triglycerides)	8.48 ± 0.58	8.14 ± 0.59
Cholesterol (mg/100 g)	444.60 ± 9.78	420.7 ± 19.34
Protein and amino acid profile		
Protein in eggs (%)	12.81 ± 0.27	12.94 ± 0.34
Protein in egg shells (%)	4.75 ± 0.05	6.69 ± 0.63*
Protein in egg yolk (%)	11.44 ± 0.67	13.56 ± 0.57
Glutamic acid (mg/g)	0.13 ± 0.01	0.19 ± 0.02
Glycine (mg/g)	4.02 ± 0.14	4.69 ± 0.33
Carotenoids		
Lutein (µg/g)	1.72 ± 0.16	3.44 ± 0.18**
Cis‐zeaxanthin (µg/g)	0.32 ± 0.04	0.60 ± 0.08*
Trans‐zeaxanthin (µg/g)	2.19 ± 0.09	2.47 ± 0.17
Total‐zeaxanthin (µg/g)	2.51 ± 0.21	3.07 ± 0.11

*Note*: CONHG: Control RIR hens; BETHG: Blessing/Biofield treated RIR hens; *p ≤ 0.05, **p ≤ 0.01, and ***p ≤ 0.001 with respect to CONHG. Data were represented as mean ± SEM (*n* = 3). Statistical analysis was performed using Student *t*‐test.

### 4.5 Assessment of Microbial Analysis on RIR Meat/Eggs/Yolk/Shells

In the meat and egg sample, *Salmonella* and *Vibrio* species were not found either in the CONHG or BETHG (Table 6).

**TABLE 6:** Microbial analysis of both control and biofield energy‐treated meat, eggs, egg yolk and egg shells.

Parameter	CONHG	BETHG
Meat		
*Pseudomonas* species (cfu/g)	< 10	< 10
*Staphylococcus aureus* (cfu/g)	< 10	< 10
*Salmonella* species (per 25 g)	Not detected	Not detected
*Vibrio* spp. (per 25 g)	Not detected	Not detected
Egg		
*Staphylococcus aureus* (cfu/g)	< 10	< 10
*Salmonella* species (per 25 g)	Not detected	Not detected
*Listeria* species (per 25 g)	Not detected	Not detected
*Vibrio* spp. (per 25 g)	Not detected	Not detected
Egg yolk		
*Pseudomonas* species (cfu/g)	< 10	< 10
*Enterobacteriaceae* (cfu/g)	< 10	< 10
Total Coliforms (cfu/g)	< 10	< 10
*E. coli* (cfu/g)	< 10	< 10
Egg shell		
*Enterobacteriaceae* (cfu/g)	< 10	< 10
Total coliforms (cfu/g)	< 10	< 10
*E. coli* (cfu/g)	< 10	< 10

Abbreviation: CFU, colony forming unit.

### 4.6 Sensory/Organoleptic Evaluation of Meat and Eggs

All the sensory parameters were significantly (*p* ≤ 0.001) improved in the BETHG compared to the CONHG in both meat and eggs (Table 7).

**TABLE 7:** Assessment of sensory characteristics of Rhode Island Red meat and eggs by consumers.

Parameter	CONHG	BETHG
Meat		
Colour	4.80 ± 0.19	8.05 ± 0.05***
Flavour/aroma/smell	4.95 ± 0.23	8.25 ± 0.10***
Taste	5.00 ± 0.19	8.25 ± 0.10***
Tenderness	5.10 ± 0.19	8.20 ± 0.09***
Juiciness	4.95 ± 0.27	8.10 ± 0.07***
Acceptability/overall quality	5.00 ± 0.25	8.25 ± 0.10***
Eggs		
Colour	5.65 ± 0.24	8.10 ± 0.18***
Flavour/aroma/smell	5.25 ± 0.22	7.70 ± 0.22***
Taste	5.15 ± 0.22	7.65 ± 0.15***
Mouth feel	5.90 ± 0.24	8.55 ± 0.11***
Texture	5.95 ± 0.25	8.50 ± 0.11***
Overall acceptability	6.20 ± 0.21	8.85 ± 0.08***

*Note*: CONHG: Control RIR hens; BETHG: Blessing/Biofield treated RIR hens; Consumer number (*n*) = 20; ***p ≤ 0.001 with respect to CONHG. Data were represented as mean ± SEM (*n* = 20). Statistical analysis was performed using Student *t*‐test.

## 5 Discussion

Poultry plays a significant role in global meat and egg production. With rising standards of community welfare, there is increasing demand for poultry meat/eggs that are not only tender, affordable and easily available but also healthy, safe and free from antibiotic residues. Hen's meat and eggs are a key source of animal protein for people around the world. However, the rising cost of chicken feed ingredients has led to an increase in chicken prices. This underscores the urgent need for cost‐effective and sustainable solutions that enable organic poultry farmers to increase production while simultaneously improving nutritional content and minimising the use of low‐cost inputs. To achieve this milestone, farmers can adopt feeding strategies and also explore alternative methods that effectively optimise chicken health, growth, weight, egg quality and nutritional composition. Numerous studies have shown that specific dietary supplement systems can significantly improve growth performance in poultry (Tovar‐Ramírez et al. 2025; Zhao et al. 2024). Religious traditions across the world display beliefs in healing through blessings and prayer (Bearon and Koenig 1990). The healing powers of blessing/prayer have been examined in randomised controlled clinical trials (Masters 2005; Teut et al. 2014). Blessing or prayer has been reported to improve outcomes in humans as well as nonhuman species, such as microbes, mice, rats and dogs (Patil et al. 2023; M. K. Trivedi et al. 2024c; M. K. Trivedi et al. 2021). Different types of blessings and prayers, including physical presence and long‐distance blessings, have been shown to result in psychological and biological changes that are either directly or potentially associated with improved health (Masters 2005; Maltby et al. 1999).

The biofield‐treated RIR hens laid bigger, heavier and denser eggs than the untreated RIR hens. The eggs from the biofield treatment hens (BETHG) did not show any eggy or sulphuric smell before and after cooking. After cooking, the BETHG eggs were found to have a very creamy, rich texture and were very tasty, which remained in the mouth for more than 5 min, compared to the CONHG eggs. The white part of the boiled eggs was not rubbery at all and melted in the mouth, and the yolk was light in colour and very creamy, rich and moist (Table 7). The egg was not chewy at all, even if you overcook it. It had a sweetness that required no additional seasoning. The scrambled egg was so creamy that it did not need a lot of cream or butter during preparation. The shell of the egg was very thick hard, and the membrane inside the shell was also very thick, strong and rubbery. Based on the consumers feedback, no bloating, acidity, burning sensation, or discomfort was found after eating the biofield‐treated eggs, and also improved mood and digestion (data not shown). There has been considerable research on the effects of Trivedi Effect on human health and well‐being, including their promotion of mental relaxation, quality of life, and social and personal relationships, as well as their alleviation of mental anguish, stress, depression and anxiety (M. K. Trivedi et al. 2024b).

Egg yolk possesses an excellent growth medium for contaminating microorganisms. Microorganisms can invade eggs through barriers like egg shells and the white protein layer to reach the yolk, where they can multiply and spoil the eggs (Al‐Bahry et al. 2012). Egg white layer (avidin) was less prone to microbial contamination due to the presence of lysozyme enzyme and alkaline pH, which prevent microbes’ multiplication (Guyot et al. 2016). A fresh‐laid egg is normally devoid of contamination; however, just after oviposition, the shell surface becomes contaminated with various pathogenic microorganisms (Damena et al. 2022). In this study, the authors observed that microbial load was within the reference ranges in both meat and eggs of the CONHG and BETHG (Table 6).

The study findings indicated that biofield energy treatment (Trivedi Effect) on egg laying (RIR) hens exhibited higher levels of nutritional components in both meat and eggs, such as vitamins, minerals, amino acids and fatty acids contents and egg morphology, compared to the untreated/unblessed RIR hens (CONHG). The observed differences in egg morphology and nutritional composition between the two groups highlight the importance of blessing therapy in poultry farming.

The blessing energy treatment has proven to be more cost‐effective in poultry farming, resulting in improved quality of chicken eggs and meat. Biofield energy medicine leverages the body's energy to enhance health, and its principles can be understood through quantum physics. Living organisms respond to low levels of non‐ionising electromagnetic fields, affecting cellular functions in hens. The “biofield” describes the energy surrounding and permeating the body, including measurable electromagnetic energy, and it's similar to Qi (Rein 2004; Wnuk and Bernard 2001). In this study, the positive effects of blessing/biofield energy treatment by a practitioner (Trivedi Effect) may arise from an energy channel transmitted to the hens. These outcomes may result from thought transmission with the universal consciousness energy (Carrol 2012), as explained by quantum entanglement (M. K. Trivedi et al. 2024a; Jana et al. 2024), and bioresonance mechanism (Muresan et al. 2022). The treatment's effects are linked to the healer's quantum energy transmission, enabling quantum‐level spiritual energy healing (Jana et al. 2024).

### 5.1 Limitations

Apart from the positive outcomes of this study, a few limitations are anticipated, such as a small sample size, a short duration of data collection period, and a single geographical location Texas, USA, which limited its generalisability. Future studies encompassing larger sample sizes, long‐term data collection and diverse locations would provide more robust and representative results.

## 6 Conclusion

Overall, the blessing (biofield) energy treatment was an efficient, cost‐effective alternative strategy that significantly improved egg‐laying rate, egg morphology and the nutritional content of meat and eggs from 28‐week‐old RIR laying hens. Briefly, the Trivedi Effect increased edible meat weight, vitamins (D_3_, B_9_ and B_12_), minerals (Fe, Zn, Cu and Na), amino acids (glutamine, serine and taurine), sensory parameters and many vital unsaturated MUFAs/PUFAs in the meat sample. Besides, egg morphological parameters (yolk weight, yolk height and egg laying rate), vitamins (D_3_, A, E, B_7_ and B_9_), minerals (Fe, Zn, P, Se and Ca), amino acids, fatty acid profiles, sensory parameters and some carotenoids (lutein and zeaxanthin) were also increased in the egg sample. Moreover, the protein content of egg yolk and egg shells was improved in the blessing treatment group compared to the untreated eggs. These findings have practical implications for the poultry industry.

## Author Contributions

**Mahendra Kumar Trivedi**: conceptualization, supervision, interpretation, writing – original draft preparation. **Vaibhav Rajan Parulkar**: design, planning, execution, monitoring, data collection, writing – review and editing. **Dahryn Trivedi**: project co‐ordinator, supervision, monitoring, data collection, visualization and writing. **Alice Branton**: writing – review and editing. **Sambhu Mondal**: data analysis, interpretation, and writing. **Snehasis Jana**: data analysis, interpretation, and writing – review and editing.

## Funding

This pilot study was funded by Mr. James Jeffery Peoples, Grow Group LP, FL, USA.

## Ethics Statement

Ethical review and approval were not obtained from the Animal Care and Use Committee for this study because the study did not reach the threshold for submission to the local ethics and welfare committee. The animals were cared for in accordance with the standard guide for the Care and Use of Agricultural Animals in Research and Teaching. The owner of the Rhode Island Red laying hens described in this study provided informed consent for the study procedures, blessing/biofield energy (non‐invasive and non‐pharmacological) treatment and use of poultry research data such as flock size, feed consumption data, egg production numbers, mortality rates, eggs and meat quality‐related parameters findings for research and publication purposes.

## Conflicts of Interest

MKT, AB and DT were employed by Trivedi Global, Inc. VRP was employed by Divine Connection International. Authors SM and SJ were employed by Trivedi Science Research Laboratory Pvt. Ltd. The authors declare that the research was conducted in the absence of any commercial or financial relationships that could be construed as a potential conflict of interest.

## Data Availability

The data that support the findings of this study are available from the corresponding author upon reasonable request.
